# Sex Differences in Muscle–Respiratory Function Relationship in Lung Transplant Patients: A Longitudinal Study

**DOI:** 10.1002/jcsm.70244

**Published:** 2026-03-05

**Authors:** Chiara Ceolin, Agnese Alessi, Anna Citron, Monica Loy, Mario Virgilio Papa, Carlotta Andaloro, Bruno Micael Zanforlini, Maria Devita, Sara Bertolino, Sara Gonnelli, Daniele Michele Seccia, Anna Bertocco, Federico Rea, Giuseppe Sergi, Marina De Rui

**Affiliations:** ^1^ Department of Medicine (DIMED) University of Padua Padua Italy; ^2^ Geriatric Division University Hospital of Padua Padua Italy; ^3^ Department of Neurobiology, Care Sciences and Society Karolinska Institutet and Stockholm University, Aging Research Center Stockholm Sweden; ^4^ Department of Cardiac, Thoracic and Vascular Sciences and Public Health, Thoracic Surgery Unit University of Padua Padua Italy; ^5^ Department of General Psychology (DPG) University of Padua Padua Italy

**Keywords:** COPD, cystic fibrosis, lung transplant, sarcopenia, vertebral fractures

## Abstract

**Background:**

Lung transplant recipients are at increased risk of sarcopenia and osteoporosis, which may negatively influence respiratory outcomes. Although muscle health is known to affect lung function, little is known about the long‐term interplay between muscle parameters and pulmonary volumes, especially across sexes. The objective of this study is to evaluate the longitudinal relationship between muscle mass and strength and respiratory function in lung transplant patients, with sex‐specific analysis.

**Methods:**

This prospective cohort included three assessments (baseline ≥ 3 months after transplant, ~1 year and 2–3 years). The primary outcome was the longitudinal change in pulmonary function (VC, FVC, FEV1 and TLC) in relation to appendicular skeletal muscle mass index (ASMMI) and handgrip strength (HGS). Associations at baseline were tested with multivariable linear regression. Analyses were performed with linear mixed‐effects models (LMM) including random intercepts for subject, time as a fixed effect and interactions between time and muscle parameters, adjusted for age, ADL, corticosteroid dose, vertebral fractures, osteoporosis, comorbidities and time since transplant.

**Results:**

We studied 155 recipients (43.2% women, age 48.7 ± 13.3 years). Primary indications were cystic fibrosis (30.1%), restrictive (22.2%), obstructive (15.7%), miscellaneous (26.8%) and vascular diseases (5.2%). At baseline, HGS was independently associated with higher VC (*R* ^2^: 0.63, β = 0.35, *p* = 0.001 in women; *R* ^2^: 0.58, β = 0.16, *p* < 0.001 in men) and FEV1 (*R* ^2^: 0.51, β = 0.08, *p* = 0.020 in women; *R* ^2^: 0.57, β = 0.19, *p* = 0.009 in men). ASMMI was independently associated with VC in both sexes (women: *R* ^2^: 0.58, β = 0.31, *p* = 0.003; men: *R* ^2^: 0.40, β = 0.16, *p* = 0.023). Longitudinally, LMMs showed that higher HGS was associated with more favourable trajectories of pulmonary function over follow‐up. Specifically, among women with restrictive disease, lower ASMMI predicted higher FEV1 (β = −4.95, 95% CI −6.93 to −2.97, *p* = 0.007) and higher TLC (β = −2.22, 95% CI −4.56 to −1.12, *p* = 0.04) over time. In women with cystic fibrosis, stronger HGS was associated with improved TLC (β = 0.38, *p* = 0.04). All associations persisted after full adjustment.

**Conclusion:**

Muscle mass and strength are associated with lung function after lung transplantation. These findings underscore the clinical importance of muscle health and support its integration into post‐transplant management.

## 1 Introduction

Lung transplantation is a worldwide therapeutic option for the treatment of numerous end‐stage lung diseases. The primary indications for this procedure include lung fibrosis, chronic obstructive pulmonary disease (COPD) and cystic fibrosis (CF) [1].

Conditions such as chronic respiratory failure, physical inactivity and corticosteroid therapy in the post‐transplant immunosuppressive regimen can increase the risk of fracture, as a result of the reduction in bone mass and quality [2], disability and mortality [3]. Moreover, inflammatory status and oxidative stress are common in the immediate post‐lung transplantation period [4], and can contribute to the loss of lean mass. For example, in COPD patients, sarcopenia rates (i.e., the loss of muscle strength and mass) range between 10% and 25% [5]. Osteoporosis and sarcopenia could, therefore, be common conditions in transplant recipients and might limit the beneficial effects of the transplant itself; vertebral fractures can lead to thoracic kyphosis, which negatively impacts lung volume [6, S1]. This is particularly important in CF patients, who undergo transplantation at a young age, and whom previous studies have shown to be at risk of bone disease, defined by low bone mineral density (BMD), and at an increased risk of fragility fractures [7, S2].

Pulmonary performance can be influenced by body composition parameters in both healthy individuals and patients with chronic lung disease, although direct comparisons between these groups remain limited [8, 9]. In healthy adults, handgrip strength (HGS) is independently associated with spirometric indices, typically with higher FEV_1_ and FVC per SD increase in HGS after adjustment for age, sex and body size [10]. For example, Han et al. reported significant positive associations of HGS with FEV_1_ and FVC in both men and women, independent of confounders, and similar findings have been observed in other cohorts [11]. In COPD cohorts, lower HGS tracks with lower FEV_1_ and worse clinical status; in interstitial lung disease, disease severity relates to reduced grip strength and poorer functional metrics [12, 13]. However, due to the heterogeneity of diagnostic criteria for sarcopenia, few studies have specifically examined the impact of sarcopenia—European Working Group on Sarcopenia in Older People (EWGSOP) original consensus in 2010 [14] and its revised 2019 version (EWGSOP2) [15]—on respiratory performance in lung transplant recipients. Nonetheless, the shared hypothesis is that both qualitative and quantitative muscle alterations, resulting from chronic disease, medications and lifestyle habits, can negatively affect lung performance. However, there is still a significant lack of research that longitudinally investigates the effects of muscle parameters, such as muscle mass and muscle strength, as well as other muscle‐related variables, on lung function over time.

Given these premises and the gaps in the current literature, this study has two primary objectives. The first aim is to characterize lung transplant recipients in terms of osteo‐muscular parameters, including the presence of osteoporosis, muscle mass and muscle strength, and to evaluate the impact of muscle parameters on respiratory function. The second aim is to determine whether changes in muscle parameters influence lung volumes over time.

## 2 Materials and Methods

### 2.1 Study Design

A longitudinal study was conducted on patients who underwent lung transplantation. The patients were enrolled as scheduled post‐transplant admissions by the Thoracic Surgery Unit (UOC) at the Padua University Hospital. Subsequently, they underwent a specialized evaluation at the Geriatric Department within the same hospital.

### 2.2 Participants

Inclusion criteria were age > 20 years and having undergone a single or double lung transplant. Exclusion criteria were (i) active neoplastic disease or positive screening for previous neoplasms within the last 5 years; (ii) history of heart–lung transplant; (iii) clinically unstable conditions (e.g., fever, worsening respiratory symptoms, recent changes in usual therapy or hospitalization for respiratory issues within 30 days before the evaluation); (iv) inability to perform spirometry or physical performance tests; and (v) lung transplant performed less than 3 months prior to enrolment.

The patients were assessed at three distinct time points: an initial evaluation conducted at least 3 months after the transplant, followed by two subsequent follow‐ups at 1 year and 2–3 years after the initial evaluation.

### 2.3 Ethical Approval

The study was conducted in strict adherence to the ethics statement of the International Society for Heart and Lung Transplantation (ISHLT). The study protocol received approval from the local ethics committee (Comitato Etico di Padova, number 0014675) and complied with the guidelines outlined in the Declaration of Helsinki. Each participant provided written informed consent to participate in the study.

### 2.4 Study Variables

Each participant underwent a clinical and functional assessment, both at baseline and during the two follow‐ups, carried out by qualified medical personnel. This assessment included the following.

#### 2.4.1 Medical History

Recent and past pathological, physiological and pharmacological history was recorded, with particular attention to corticosteroid, immunosuppressive and anti‐osteoporosis therapy, as well as previous diagnosis of osteoporosis. Comorbidities and disease severity were evaluated using the Cumulative Illness Rating Scale (CIRS) (please see reference S3).

#### 2.4.2 Functional Assessment

Functional status was evaluated based on independence in Activities of Daily Living (ADL) (please see reference S4).

#### 2.4.3 Anthropometric Measurements

Weight and height were measured to the nearest 0.1 kg and 0.1 cm using a scale and stadiometer (Seca—Germany), without shoes and in light clothing. Body mass index (BMI) was calculated by dividing weight (in kilograms) by height (in metres) squared.

*Instrumental methods:*

- Dual‐energy x‐ray absorptiometry (DEXA): Bone mineral density (BMD) was measured for each patient using dual‐energy x‐ray absorptiometry (DEXA) (Hologic QDR 4500W Inc.), acquiring scans of the proximal femur region (left or right) and lumbar spine (L1–L4) (please see references S5–S7). Body composition was then measured through a whole‐body scan, specifically evaluating fat‐free mass (FFM), fat mass (FM) and appendicular skeletal muscle mass (ASMM). The appendicular skeletal muscle mass index (ASMMI) was obtained by dividing the ASMM by height in metres squared. DEXA is considered the most reliable method for assessing muscle mass (total body FFM or ASMM) due to its simplicity and safety; the required radiation dose is minimal, and the measurements are generally more accurate than those obtained with other techniques.
- Spinal x‐rays: Each patient was screened for vertebral fractures using x‐rays taken in anteroposterior and lateral views the day before the evaluation. Vertebral fractures documented radiologically were defined according to the semi‐quantitative Genant method as reductions greater than 20% in anterior, middle or posterior vertebral height and were classified by severity as mild (20%–25%), moderate (26%–40%) or severe (> 40%) (please see reference S8). Vertebral fractures noted in medical history or identified in previous x‐rays and confirmed by radiographic reading were classified as known vertebral fractures, whereas those detected for the first time during outpatient radiographic reading were classified as unknown vertebral fractures.
- Spirometry: Spirometry was performed with a spirometer (Jaeger) calibrated according to the manufacturer's technical instructions and administered by the Respiratory Physiopathology Unit Team. The test was performed in the morning, and the best of three spirometric tests performed was considered. The spirometric parameters evaluated were tidal volume (VT), forced vital capacity (FVC), forced expiratory volume in 1 s (FEV1) and total lung capacity (TLC). These values were normalized for age, sex, weight, height and ethnicity according to international spirometric reference values and expressed as a percentage of the predicted value (% pred).

#### 2.4.4 Muscle Strength Tests

Handgrip strength (HGS) was measured on the dominant side using an electronic dynamometer (Akern DynEx). Patients were seated on a standard chair with the dominant arm resting neutrally with the elbow flexed at 90° on the table and the forearm and wrist in a natural position. They were instructed to grip the dynamometer and squeeze to their maximum strength in response to a verbal command, without making any sudden movements. Two measurements were taken on the dominant side with a 1‐min interval between trials, and the highest measurement was used for our analyses (please see reference S9).

#### 2.4.5 Biochemical Parameters

General blood tests were recorded for each patient, and the following phospho‐calcium metabolism tests performed within 3 months of the visit: serum calcium, serum phosphorus, parathyroid hormone (PTH), 25‐hydroxy‐vitamin D, CTX (C‐terminal telopeptide of Type 1 collagen), total and bone alkaline phosphatase, 24‐h urine calcium and phosphate excretion. The analyses were performed following standard procedures at the laboratory unit of the University Hospital of Padua, which has Clinical Pathology Accreditation.

### 2.5 Assessment of Osteoporosis and Sarcopenia

Osteoporosis was evaluated based on lumbar and femoral T‐score values, as well as the presence of fragility fractures, such as a proximal femur or vertebral fracture. According to the definitions provided by the World Health Organization and the International Osteoporosis Foundation (please see reference S10), four conditions were identified: A T‐score of −1.0 or higher indicated normal bone mineral density, a T‐score between −1.0 and −2.5 indicated osteopenia, and osteoporosis was defined by a T‐score of −2.5 or lower. In accordance with the subsequent refinement proposed by Ferrari et al. (please see reference S11), patients with a T‐score of −2.5 or lower and one or more fragility fractures were classified as having severe osteoporosis.

Sarcopenia was diagnosed based on muscle strength, mass and performance, following the 2019 European Consensus criteria [15]. Values of HGS below 16 kg for women and 27 kg for men or chair stand test scores > 15 s suggested probable sarcopenia and were categorized as low muscle strength. Low muscle mass was defined as ASMMI values below 5.5 kg/m^2^ for women and 7.0 kg/m^2^ for men. The contemporaneous presence of low muscle strength and low muscle mass defined confirmed sarcopenia.

### 2.6 Statistical Analyses

The characteristics of the studied sample were expressed as mean and standard deviation (SD) for normally distributed continuous quantitative variables, as median (interquartile range) for non‐normally distributed variables or counts and percentages for categorical variables. The normality of the distributions for continuous quantitative variables was assessed using the Shapiro–Wilk test. The characteristics of the study participants and the results of the tests were compared based on sex, underlying respiratory condition, presence of osteoporosis and sarcopenia using the Mann–Whitney and Kruskal–Wallis tests for quantitative variables and Pearson's chi‐square test for categorical variables.

To determine whether muscle parameters (mass and strength) were independent predictors of VC, FVC, FEV1 and TLC at baseline, a multivariate linear regression analysis was conducted, stratifying by sex and adjusting for age, functional abilities, steroid dosage, osteoporosis, presence of vertebral fractures, CIRS‐CI and time since transplantation. To evaluate whether pulmonary function changed over time in relation to muscle parameters, we used linear mixed‐effects models (LMMs) with repeated measures. Each participant contributed multiple observations; subject was modelled as a random intercept, whereas time was included as a fixed effect. ASMMI and HGS were modelled as fixed effects, and, when supported by convergence, random slopes for time were also considered. Interaction terms (time × ASMMI and time × HGS) were included to assess whether trajectories differed by muscle status. Models were stratified by sex and additionally by pre‐transplant diagnosis. Covariates included age, ADL, corticosteroid dose, vertebral fractures, osteoporosis, comorbidity burden (CIRS‐CI) and time since transplant.

Statistical tests were deemed significant with a *p*‐value < 0.05. All analyses were performed using the Statistical Package for the Social Sciences Version 29.0 (SPSS, Armonk, NY: IBM Corp).

## 3 Results

### 3.1 Baseline Characteristics

Table 1 summarizes the characteristics of the study population (*n* = 155; 67 women, 43.2%). The mean age was 48.7 ± 13.3 years (range 21–72), with women being significantly younger than men (*p* < 0.001). The main indications for lung transplantation were CF (30.1%), restrictive lung diseases (22.2%), obstructive lung diseases (15.7%), miscellaneous conditions (26.8%) and vascular diseases (5.2%). CF was more frequent among women (42.4% vs. 20.7%), whereas obstructive lung diseases were more common in men (21.8% vs. 7.6%; both *p* = 0.01). On average, participants were assessed 40 months after their transplantation.

**TABLE 1:** Descriptive characteristics of the total sample and by gender at baseline.

Variable	Total (*n* = 155)	Women (*n* = 67)	Men (*n* = 88)	*p*
Age (years), mean ± SD	48.7 ± 13.3	44.6 ± 14.3	51.9 ± 11.5	< 0.001
BMI (kg/m^2^), mean ± SD	22.9 ± 4.03	21.36 ± 3.46	24.2 ± 3.99	0.200
ADL, mean ± SD	5.84 ± 0.62	5.88 ± 0.48	5.81 ± 0.71	0.46
Primary condition, *n* (%)				0.01
CF	46 (30.1%)	28 (42.4%)	18 (20.7%)	
Restrictive lung diseases	34 (22.2%)	16 (24.2%)	18 (20.7%)	
Obstructive lung diseases	24 (15.7%)	5 (7.6%)	19 (21.8%)	
Miscellaneous	41 (26.8%)	13 (19.7%)	28 (32.2%)	
Vascular diseases	8 (5.2%)	4 (4.6%)	4 (6.1%)	
Time since transplant (months), mean ± SD	40.01 ± 80.71	40.94 ± 52.76	39.31 ± 97.05	< 0.001
Total medications taken, mean ± SD	15.05 ± 3.86	14.87 ± 3.88	15.18 ± 3.87	0.007
Duration of corticosteroid therapy (months), mean ± SD	30.19 ± 47.98	38.51 ± 61.60	23.84 ± 33.12	< 0.001
CIRS‐CI, mean ± SD	4.05 ± 1.67	4.00 ± 1.52	4.08 ± 1.79	< 0.001
Pulmonary volumes, mean ± SD				
VC (% predicted)	78.5 ± 20.27	82.0 ± 20.61	73.0 ± 19.49	0.094
FVC (% predicted)	75.94 ± 20.52	78.40 ± 20.13	73.97 ± 20.74	0.013
FEV1 (% predicted)	78.0 ± 21.64	78.0 ± 20.81	77.0 ± 22.26	0.084
FEV1/FVC ratio (%)	84.20 ± 9.04	85.15 ± 8.57	83.41 ± 9.40	0.30
TLC (% predicted)	78.50 ± 17.07	83.0 ± 15.48	71.0 ± 17.00	0.200
Muscle variables				
Handgrip strength (kg), mean ± SD	26.49 ± 9.57	20.73 ± 5.90	30.86 ± 9.52	0.016
ASMMI (kg/m^2^), mean ± SD	6.51 ± 1.22	5.71 ± 0.97	7.12 ± 1.02	< 0.001
Low muscle strength, *n* (%)	44 (28.8%)	13 (8.4%)	31 (20.1)	0.03
Low muscle mass, *n* (%)	68 (44.4%)	25 (16.2%)	43 (27.9%)	0.16
Sarcopenia prevalence, *n* (%)	29 (19.1%)	10 (15.2%)	19 (22.1%)	0.06

*Note:* Descriptive statistics are presented as mean (standard deviation), median (25th–75th percentile) or count (percentages). Pulmonary volumes are % predicted; FEV_1_/FVC is reported as %.

Abbreviations: ADL, Activities of Daily Living; ASMMI, appendicular skeletal muscle mass index; BMI, body mass index; CIRS‐CI, Cumulative Illness Rating Scale—Comorbidity Index; CF, Cystic Fibrosis; FEV1, forced expiratory volume in 1 s; FEV_1_/FVC, ratio of FEV_1_ to FVC; FVC, forced vital capacity; TLC, total lung capacity; VC, vital capacity.

The majority of patients (98.70%) were on immunosuppressive therapy. Of these, 11.7% were on monotherapy, whereas the remaining 87% were receiving a combination of drugs. The most frequently used medications in these combinations included cyclosporine (31.2%), tacrolimus (63.6%), azathioprine (1.3%), everolimus (14.9%) and mycophenolate (79.2%). Additionally, 99.4% of patients were treated with glucocorticoids. The median duration of continuous corticosteroid therapy was significantly longer in patients with CF (*p* < 0.001).

When examining pulmonary function, there were no significant differences between men and women, except for FVC, which was lower in men [73.97 (20.74) vs. 78.40 (20.13), *p* = 0.013]. Men tended to have lower levels of muscle strength and muscle mass, although the overall prevalence of sarcopenia did not differ significantly between the sexes. Patients with restrictive lung diseases more often exhibited reduced muscle strength, although there were no significant differences in the rates of reduced muscle mass and sarcopenia across different respiratory conditions (data not shown).

Biochemical analyses revealed that 48% of patients had vitamin D deficiency (with levels below 50 nmol/L). Furthermore, 30 patients presented with hypocalcaemia (serum calcium levels below 2.2 mmol/L), and 55 patients had urinary calcium levels below the normal range (< 2.5 mmol/24 h) (data not shown).

Densitometric values and osteoporosis prevalence (both pre‐ and post‐transplant) are reported in Table 2. Before transplantation, 21.9% of patients were diagnosed with osteoporosis, with a slightly higher prevalence in men than in women (23.9% versus 19.4%, *p* = 0.01). Additionally, 18.7% of patients were receiving anti‐resorptive therapy, with no significant differences between sexes. Eighteen patients already had known vertebral fractures. Fracture prevalence differed according to some immunosuppressive agents: It was higher among cyclosporine users (35.4% vs. 13.2%; χ^2^ = 10.14, *p* = 0.001) and lower among tacrolimus users (13.3% vs. 32.1%; χ^2^ = 7.90, *p* = 0.005). No significant differences were observed for everolimus (*p* = 0.18) or mycophenolate (*p* = 0.21), whereas azathioprine was used in only two patients, both with events (*p* = 0.039). After our assessment, it was found that 18.7% of patients had osteopenia and 52.9% had osteoporosis; moreover, men had a higher incidence of vertebral fractures. Overall, 52 patients (33.5%) had at least one vertebral fracture. The percentage of patients treated with anti‐resorptive therapy increased to 71% following the geriatric evaluation, and all patients received calcium and vitamin D supplementation. Considering the underlying lung disease, osteoporosis was more common among patients with CF and restrictive lung disease. However, approximately 46% of patients with obstructive lung disease had at least one vertebral fracture (*p* = 0.04).

**TABLE 2:** Densitometric values and osteoporosis prevalence of the total sample and by sex at baseline.

Variable	Total (*n* = 155)	Women (*n* = 67)	Men (*n* = 88)	*p*
Pre‐transplant evaluation, *n* (%)				
Vitamin D/calcium supplementation	102 (62.5%)	48 (72.7%)	54 (61.4%)	0.09
Anti‐resorptive therapy	29 (18.7%)	9 (13.4%)	20 (22.7%)	0.10
Anabolic therapy	2 (1.3%)	0	2 (2.3%)	0.45
Osteoporosis diagnosis	34 (21.9%)	13 (19.4%)	21 (23.9%)	0.01
Post‐transplant evaluation				
Densitometric values, mean ± SD				
T‐score lumbar	−1.71 ± 1.41	−1.73 ± 1.36	−1.69 ± 1.46	0.86
T‐score femur neck	−1.92 ± 1.09	−2.22 ± 0.94	−1.70 ± 1.14	< 0.001
T‐score total hip	−1.53 ± 1.01	−1.91 ± 0.94	−1.25 ± 0.98	< 0.001
BMD lumbar	0.92 ± 0.49	0.94 ± 0.73	0.90 ± 0.16	0.58
BMD femur neck	0.92 ± 0.49	0.62 ± 0.13	0.69 ± 0.15	< 0.001
BMD total hip	0.77 ± 0.18	0.71 ± 0.14	0.83 ± 0.17	< 0.001
Diagnosis, *n* (%)				0.10
Osteopenia	29 (18.7%)	9 (13.4%)	20 (22.7%)	
Osteoporosis	82 (52.9%)	25 (37.3%)	57 (64.7%)	
At least one vertebral fracture, *n* (%)	52 (33.5%)	14 (20.9%)	38 (43.2%)	0.005
Anti‐resorptive therapy, *n* (%)	109 (70.3%)	41 (61.2%)	68 (77.3%)	0.003
Anabolic therapy, *n* (%)	11 (7.1%)	2 (3.0%)	9 (10.2%)	0.003

*Note:* Descriptive statistics are presented as mean (standard deviation), median (25th–75th percentile) or count (percentages).

Abbreviation: BMD, bone mineral density.

### 3.2 Relationship Between Lung Volumes and Muscle Parameters at Baseline

**FIGURE 1:**
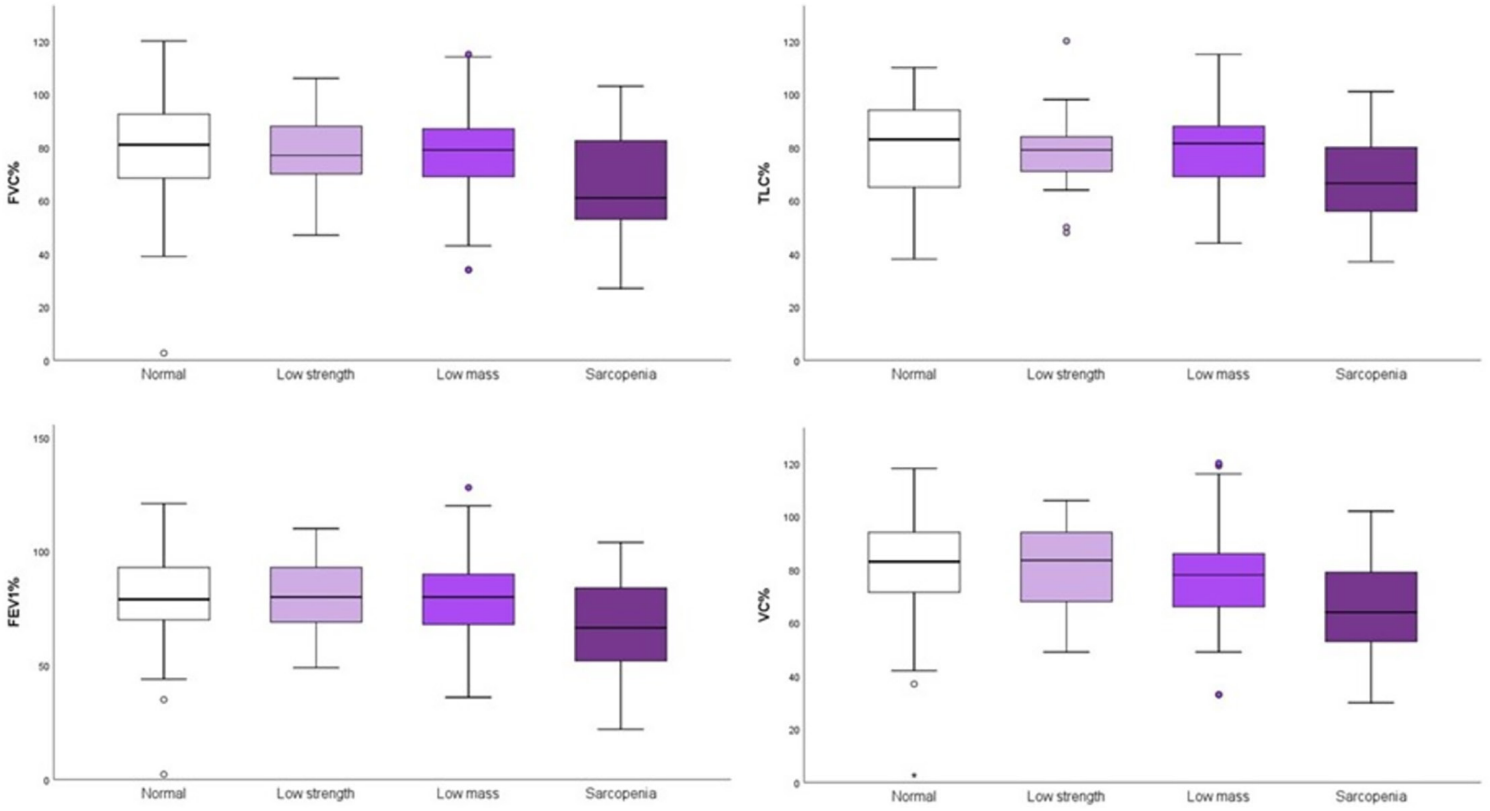
Spirometric parameters according to muscle variables.

At baseline, spirometric volumes differed according to muscle status (Figure 1). Patients with low muscle strength, low muscle mass and sarcopenia showed progressively lower values of VC, FVC, FEV1 and TLC compared with those with normal muscle parameters. The lowest spirometric values were consistently observed in patients with sarcopenia.

In multivariable linear regression analyses stratified by sex (Table 3), HGS was independently associated with higher VC in both men (β = 0.16, *p* < 0.001) and women (β = 0.35, *p* = 0.001). HGS was also associated with higher FVC in men (β = 0.37, *p* < 0.001) and women (β = 0.33, *p* = 0.001) and with higher FEV1 in both sexes (men: β = 0.19, *p* = 0.009; women: β = 0.08, *p* = 0.020). In men, HGS was additionally associated with higher TLC (β = 0.23, *p* = 0.005). ASMMI was independently associated with VC in both men (β = 0.16, *p* = 0.023) and women (β = 0.31, *p* = 0.003) and with FVC in men (β = 0.16, *p* = 0.040). All associations remained significant after adjustment for age, functional status, corticosteroid dose, osteoporosis, vertebral fractures, comorbidity burden and time since transplantation.

**TABLE 3:** Linear regression analysis for predictors of lung volumes, stratified by gender.

	Men		Women	
Variable	ASMMI	HGS	ASMMI	HGS
VC	*R* ^2^: 0.40 β: 0.16, *p* = 0.023	*R* ^2^: 0.58 β: 0.16, *p* < 0.001	R^2^: 0.58 β: 0.31, *p* = 0.003	*R* ^2^: 0.63 β: 0.35, *p* = 0.001
FVC	*R* ^2^: 0.40 β: 0.16, *p* = 0.040	*R* ^2^: 0.60 β: 0.37, *p* < 0.001	—	*R* ^2^: 0.60 β: 0.33, *p* = 0.001
FEV1	—	*R* ^2^: 0.57 β: 0.19, *p* = 0.009	—	*R* ^2^: 0.51 β: 0.08, *p* = 0.020
TLC	—	*R* ^2^: 0.54 β: 0.23, *p* = 0.005	—	—

*Note:* The table displays the *R* ^2^ values, standardized regression coefficients (β), and *p*‐values for the linear regression models.

Abbreviations: ASMMI, appendicular skeletal muscle mass index; FEV1, forced expiratory volume in 1 s; FVC, forced vital capacity; HGS, handgrip strength test; TLC, total lung capacity; VC, tidal volume.

### 3.3 Relationship Between Lung Volumes and Muscle Parameters Over Time

The temporal distribution of pulmonary function parameters across follow‐up is illustrated in Figure S1, showing VC, FVC, FEV1 and TLC at 1‐, 2‐ and 3‐year follow‐up. Table 4 presents the results from the longitudinal mixed linear models, which account for repeated measures within subjects, used to assess the association between lung volumes and muscle parameters over time. An inverse relationship was found between ASMMI and FEV1 [β: −4.95, 95% CI (−6.93; 2.97), *p* = 0.007] as well as TLC [β: −2.22, 95% CI (−4.56; −1.12), *p* = 0.04] in women with restrictive lung disease. These associations remained significant after adjusting for age, functional abilities, steroid dosage, osteoporosis, presence of vertebral fractures, CIRS‐CI and time since transplantation. In patients with CF, TLC was significantly associated with muscle strength in women [β: 0.38, 95% CI (−0.07; 0.84), *p* = 0.04] (please see Table S1), also after adjustment for covariates.

**TABLE 4:** Linear mixed models for changes in lung volumes over time in patients with restrictive lung disease, stratified by gender.

Variable	Men			Women		
	Model 1	Model 2	Model 3	Model 1	Model 2	Model 3
	β coefficient (95% CI), *p*	β coefficient (95% CI), *p*	β coefficient (95% CI), *p*	β coefficient (95% CI), *p*	β coefficient (95% CI), *p*	β coefficient (95% CI), *p*
VC						
HGS × time	−0.09 (−1.04; 0.84), *p* = 0.79	−0.31 (−1.87; 1.24), *p* = 0.67	0.09 (−2.10; 2.29), *p* = 0.93	−0.17 (−0.82; 0.48), *p* = 0.51	−0.25 (−0.88; 0.39), *p* = 0.31	−0.37 (−1.57; 0.83), *p* = 0.20
ASMMI × time	−0.001 (−3.12; 3.12), *p* = 0.99	−0.40 (−4.80; 4.01), *p* = 0.84	1.40 (−4.44; 7.24), *p* = 0.61	−1.17 (−4.85; 2.51), *p* = 0.43	−0.79 (−5.10; 3.52), *p* = 0.61	−1.07 (−7.28; 5.14), *p* = 0.58
FVC						
HGS × time	−0.35 (−1.39; 0.68), *p* = 0.41	−0.53 (−2.22; 1.17), *p* = 0.51	−0.07 (−2.60; 2.45), *p* = 0.95	−0.05 (−0.88; 0.78), *p* = 0.88	−0.18 (−0.89; 0.52), *p* = 0.48	−0.11 (−1.12; 0.89), *p* = 0.73
ASMMI × time	−0.57 (−4.16; 3.01), *p* = 0.68	−0.98 (−5.80; 3.85), *p* = 0.66	1.01 (−5.66; 7.68), *p* = 0.74	−2.78 (−5.90; 0.34), *p* = 0.07	−2.17 (−4.96; 0.63), *p* = 0.09	−2.37 (−6.52; 1.78), *p* = 0.14
FEV1						
HGS × time	−0.20 (−1.88; 1.48), *p* = 0.77	−0.08 (−2.52; 2.36), *p* = 0.94	−0.88 (−3.48; 1.70), *p* = 0.46	0.12 (−1.16; 1.40), *p* = 0.82	−0.02 (−1.33; 1.29), *p* = 0.97	0.20 (−1.22; 1.62), *p* = 0.71
ASMMI × time	0.78 (−4.80; 6.36), *p* = 0.73	0.27 (−6.71; 7.26), *p* = 0.93	−1.81 (−9.38; 5.75), *p* = 0.60	**−5.63 (−7.89; −3.37), *p* = 0.002**	**−5.12 (−6.75; −3.50), *p* = 0.002**	**−4.95 (−6.93; 2.97), *p* = 0.007**
TLC						
HGS × time	0.16 (−0.45; 0.76), *p* = 0.52	−0.18 (−1.28; 0.89), *p* = 0.70	0.07 (−1.63; 1.77), *p* = 0.93	−0.21 (−0.72; 0.30), *p* = 0.32	−0.22 (−0.88; 0.45), *p* = 0.38	−0.06 (−0.73; 0.61), *p* = 0.80
ASMMI × time	0.46 (−1.56; 2.48), *p* = 0.56	−1.03 (−3.93; 1.87), *p* = 0.46	−0.82 (−5.36; 3.63), *p* = 0.70	**−2.02 (−3.97; −0.09), *p* = 0.02**	**−2.28 (−4.34; −0.21), *p* = 0.04**	**−2.22 (−4.56; −1.12), *p* = 0.04**

*Note:* Model 1 includes age. Model 2 also includes functional capacities and steroid dosage. Model 3 includes the presence of vertebral fractures, CIRS‐CI and time since transplant. Significant results (*p* < 0.05) are shown in bold.

Abbreviations: ASMMI, appendicular skeletal muscle mass index; FEV1, forced expiratory volume in 1 s; FVC, forced vital capacity; HGS, handgrip strength test; TLC, total lung capacity; VC, tidal volume.

## 4 Discussion

This is the first study to examine over a period of 3 years the association between muscle parameters and respiratory function. Muscle mass was found to be significantly associated with lung volumes such as TLC and FEV1 in women with restrictive lung disease, whereas muscle strength was associated with TLC in patients with CF. The magnitude of these associations was modest, which is expected given the complex and multifactorial nature of lung function after lung transplantation. Nevertheless, these findings highlight the clinical relevance of muscle assessment in lung transplant recipients, even after accounting for vertebral fractures, time since transplantation and corticosteroid exposure.

In patients undergoing lung transplantation, several conditions can lead to the development of osteoporosis, largely due to the immunosuppressive therapy regimen—which includes glucocorticoids, calcineurin inhibitors, selective mTOR inhibitors, mycophenolate or azathioprine [16]. Glucocorticoids act, for example, through a direct mechanism by promoting the differentiation, activation and survival of osteoclasts [17, S12], as well as through an indirect mechanism by inhibiting the synthesis of gonadal steroids and reducing calcium transport across intestinal, renal tubular, parathyroid cell membranes and hypothalamic membranes of the pituitary–gonadal axis [18, S13]. The percentage of patients with osteoporosis in our study is consistent with previously reported data (please see references S14 and S15), particularly among people with obstructive diseases (please see references S16–S18). It is known that osteoporosis can have significant impacts on respiratory health. The altered conformation of the rib cage associated with vertebral fractures could compromise the amount of air contained in the lungs and the amount that can be forcibly exhaled after taking the deepest breath possible (please see reference S19). For this reason, patients undergoing lung transplantation are often subjected to a comprehensive osteometabolic evaluation to facilitate the early identification of this condition.

In addition to bone metabolism, muscle health is also frequently compromised in these patients. Retrospective studies conducted on lung transplant candidates documented a decrease in lean mass in patients with COPD and interstitial lung diseases and in individuals with CF (please see references S20 and S21). To date, the aetiology of muscle mass and strength loss in these patients is still not fully understood. Factors such as physical inactivity, weight loss, inflammatory cytokines, inadequate caloric/protein intake, oxidative stress and reduced blood flow to the muscles are certainly common characteristics associated with both loss of skeletal muscle and diseases that cause chronic respiratory failure, contributing to the development of so‐called secondary sarcopenia [15, 19]. Moreover, drugs like glucocorticoids, cyclosporine and tacrolimus are known for their catabolic effects on muscles, contributing to reduced protein synthesis and increased muscle degradation [16]. In our study, 29% of participants exhibited a decrease in muscle strength, whereas 44% showed a reduction in muscle mass, results consistent with previous studies [20]. We observed that the reduction in muscle strength was significantly greater in men, whereas there were no significant sex‐differences regarding muscle mass. These findings contrast with those of Nikkuni et al. [21], who reported a higher prevalence of sarcopenia in women post‐transplant. This discrepancy may be explained by the generally older age of the male sample compared to the female sample in our study. The literature agrees that good muscle performance is associated with better spirometric parameters: Feng et al. found that lean mass is positively correlated with FVC and FEV1 in healthy adults [22]. Son et al. documented that FVC and FEV1 increase progressively with improved grip strength in older patients [23], whereas Attaway et al. observed that pectoral muscle cross‐sectional area is positively associated with FEV1, FVC and the FEV1/FVC ratio in COPD patients [24]. These results highlight the negative impact that loss of muscle mass can have on pulmonary function. Indeed, inspiration, a key component of the breathing process, relies on the diaphragm and inspiratory muscles, with lung volumes such as VC, FVC, FEV1 and TLC depending on this mechanism. Our results support this, showing that at baseline, muscle strength is an independent predictor of VC, FVC, FEV1 and TLC in men, whereas appendicular lean mass is an independent predictor of VC in both sexes.

Beyond the muscle–lung physiology, unmeasured factors such as habitual physical activity and participation in structured rehabilitation could favourably influence muscle strength and, indirectly, ventilatory performance. In lung transplant recipients, exercise‐based pulmonary rehabilitation consistently improves exercise capacity (e.g., 6‐min walk distance and peak VO_2_) and muscle function, with supportive evidence from systematic reviews and meta‐analyses as well as randomized and controlled studies [25, 26]. However, the content, intensity and long‐term maintenance of muscle‐focused components vary substantially across rehabilitation programmes and clinical settings. Moreover, targeted inspiratory muscle training (often integrated within rehabilitation) can augment respiratory muscle performance and further enhance functional outcomes in this population [27]. Notably, observational work shows that many recipients remain insufficiently active months after transplantation, suggesting that effective muscle‐focused rehabilitation may not be uniformly implemented or sustained and underscoring the potential for differential exposure to activity/rehabilitation to confound associations between muscle parameters and lung volumes [28]. Unfortunately, detailed data on physical activity and rehabilitation adherence were not available in our cohort; future studies incorporating these measures are warranted to disentangle their mediating and confounding effects.

A major limitation of the existing literature on these topics is that studies have predominantly focused on the immediate post‐transplant period, without providing evidence on how muscle parameters might affect respiratory volumes over time. Our study is pioneering in evaluating, over a period of 3 years, the association between muscle mass and strength and respiratory volumes. Our results demonstrate a negative relationship between appendicular lean mass and volumes such as TLC and FEV1 in women with restrictive lung diseases, even after adjusting for factors such as the presence of osteoporosis and severe osteoporosis, corticosteroid dosage and time since transplantation. Several explanations might account for these seemingly paradoxical findings. Firstly, it is plausible that pulmonary volumes improve relatively quickly after transplantation, whereas the recovery of muscle mass is a much slower process. This discrepancy could explain why improvements in pulmonary volumes do not immediately correlate with an increase in muscle mass. Another possible explanation involves compensatory mechanisms. As pulmonary function improves, patients may rely less on accessory respiratory muscles, which were previously overused. This reduction in the reliance on these muscles could lead to some degree of atrophy, as they are no longer subjected to the same level of stress. Conversely, we observed a direct association between muscle strength and TLC values in patients with CF over time. This finding has several important implications. First, because exercise performance in CF patients is often limited by pulmonary function, stronger respiratory muscles may enhance exercise tolerance, potentially helping to maintain or even improve pulmonary function over time. This suggests that interventions aimed at improving respiratory muscle strength could have significant benefits for both pulmonary function and exercise capacity [29]. Furthermore, nutritional status is crucial for supporting muscle strength [30], which is necessary for effective breathing and the maintenance of lung volumes such as TLC. Accordingly, adequate nutritional intake is considered a cornerstone of care in lung transplant patients. Current evidence and practical guidelines underline that nutritional support should begin early, particularly when oral intake cannot meet ≥ 60% of estimated energy/protein needs [31, 32]. Indeed, in lung transplantation, the metabolic burden from surgery, immunosuppression, corticosteroids and catabolic stress requires proactive nutritional strategies [33]. In clinical practice, this means formal integration of dietitians into the transplant team, scheduled reassessments of protein–calorie adequacy (especially in the first year) and prompt escalation to supplements or enteral nutrition (or additional strategies) when deficits emerge. Implementing standardized nutrition protocols across centres could help optimize muscle mass recovery, mitigate bone loss and potentially improve long‐term pulmonary–muscle–bone interactions [34]. Although the present study did not intervene nutritionally, our findings underscore that metabolic and musculoskeletal health must remain a therapeutic target alongside immunologic and pulmonary care.

Beyond these mechanistic considerations, the observed sex‐specific patterns may also reflect differences in disease distribution, muscle biology and exposure to risk‐modifying factors. In our cohort, women were more frequently affected by CF, whereas men more often presented with obstructive diseases, a distribution that can shape distinct muscle–lung trajectories and rehabilitation needs. Accordingly, the lower muscle mass observed in men in this cohort should not be interpreted as reflecting sex‐related hormonal mechanisms, as men generally exhibit greater muscle mass than women in the general population. Sex differences in respiratory physiology—including lung and airway size–function relationships and the work of breathing—are well documented and may interact with muscle status to influence spirometric volumes [35]. Importantly, hormonal influences relevant to skeletal muscle are not limited to menopause: Oestrogens modulate satellite cell activity, mitochondrial function and muscle repair, whereas androgens exert anabolic effects on muscle protein synthesis; such pathways can differentially affect muscle quantity and quality in women and men across adulthood [36, 37, 38]. In CF specifically, female sex hormones have been implicated in disease expression (e.g., effects on airway surface liquid, mucociliary clearance, infection and inflammation), potentially contributing to sex‐related differences in functional trajectories after transplant [39]. Moreover, sex differences in inspiratory muscle characteristics (e.g., fatigability profiles) have been reported and could partly modulate the coupling between appendicular muscle status and lung volumes [40].

### 4.1 Limitations and Strengths

Among the limitations of our study is the heterogeneity of the sample in terms of age, underlying pathology and time since transplantation—factors that may differentially influence both bone and muscle mass. The limited sample size within each diagnostic subgroup also prevented more refined stratified analyses, which might have provided additional insights, and the single‐centre design may limit generalizability to other settings. In addition, data on pre‐transplant body composition as well as on patients' physical activity levels and participation in rehabilitation programs were not available. A major strength of this study is the comprehensive 3‐year follow‐up, which allowed a longitudinal evaluation of the relationship between muscle parameters and lung function in lung transplant recipients. Another strength is the comprehensive clinical characterization of the cohort, including bone health, vertebral fractures, corticosteroid exposure and comorbidity burden, allowing for extensive adjustment of potential confounders.

### 4.2 Conclusions

In conclusion, our study highlights the importance of muscle mass and strength in the respiratory function of lung transplant patients. Lower appendicular lean mass is associated with high respiratory volumes in women with restrictive lung diseases, suggesting that muscle recovery may be slower compared to improvements in pulmonary function and underscoring the need for greater focus on muscle rehabilitation during the post‐operative period. On the contrary, in patients with CF, increased muscle strength is correlated with better lung volumes over time, indicating that strengthening respiratory muscles could also support pulmonary function. We hope that this study encourages further research, ideally with more homogeneous samples and more comprehensive data collection, to further explore the relationship between muscle mass and respiratory function.

## Funding

The authors have nothing to report.

## Ethics Statement

The study was conducted in strict adherence to the ethics statement of the International Society for Heart and Lung Transplantation (ISHLT). The study protocol received approval from the local ethics committee (Comitato Etico di Padova, number 0014675) and complied with the guidelines outlined in the Declaration of Helsinki.

## Consent

Informed consent was obtained from all subjects involved in the study. Written informed consent has been obtained from the patients to publish this paper.

## Conflicts of Interest

The authors declare no conflicts of interest.

## Supporting information


**Figure S1:** Violin plots showing the distribution of pulmonary function parameters over time.
**Table S1:** Linear mixed models for changes in lung volumes over time in patients with cystic fibrosis, stratified by gender.

## Data Availability

All data generated or analysed during this study are included in this published article (and its Supporting Information).
