# Correction to ‘Sex Differences in Muscle–Respiratory Function Relationship in Lung Transplant Patients: A Longitudinal Study’

**DOI:** 10.1002/jcsm.70314

**Published:** 2026-05-12

C. Ceolin , A. Alessi , A. Citron , et al., “Sex Differences in Muscle–Respiratory Function Relationship in Lung Transplant Patients: A Longitudinal Study,” Journal of Cachexia, Sarcopenia and Muscle17, no. 2 (2026): e70244, 10.1002/jcsm.70244.PMC1296134941782505

In the originally published version of the article, a number of *p*‐values in Table 1 were reported incorrectly. Specifically, the *p*‐value for BMI was given as 0.200 but should read < 0.001; for time since transplant, < 0.001 should read 0.890; for total medications taken, 0.007 should read 0.614; for duration of corticosteroid therapy, < 0.001 should read 0.061; and for CIRS‐CI, < 0.001 should read 0.770.

Incorrect Table 1:

**Table:** 

Variable	Total (*n* = 155)	Women (*n* = 67)	Men (*n* = 88)	*p*
Age (years), mean ± SD	48.7 ± 13.3	44.6 ± 14.3	51.9 ± 11.5	< 0.001
BMI (kg/m^2^), mean ± SD	22.9 ± 4.03	21.36 ± 3.46	24.2 ± 3.99	0.200
ADL, mean ± SD	5.84 ± 0.62	5.88 ± 0.48	5.81 ± 0.71	0.46
Primary condition, *n* (%)				0.01
Cystic fibrosis	46 (30.1%)	28 (42.4%)	18 (20.7%)	
Restrictive lung diseases	34 (22.2%)	16 (24.2%)	18 (20.7%)	
Obstructive lung diseases	24 (15.7%)	5 (7.6%)	19 (21.8%)	
Miscellaneous	41 (26.8%)	13 (19.7%)	28 (32.2%)	
Vascular diseases	8 (5.2%)	4 (4.6%)	4 (6.1%)	
Time since transplant (months), mean ± SD	40.01 ± 80.71	40.94 ± 52.76	39.31 ± 97.05	< 0.001
Total medications taken, mean ± SD	15.05 ± 3.86	14.87 ± 3.88	15.18 ± 3.87	0.007
Duration of corticosteroid therapy (months), mean ± SD	30.19 ± 47.98	38.51 ± 61.60	23.84 ± 33.12	< 0.001
CIRS‐CI, mean ± SD	4.05 ± 1.67	4.00 ± 1.52	4.08 ± 1.79	< 0.001
Pulmonary volumes, mean ± SD				
VC (% predicted)	78.5 ± 20.27	82.0 ± 20.61	73.0 ± 19.49	0.094
FVC (% predicted)	75.94 ± 20.52	78.40 ± 20.13	73.97 ± 20.74	0.013
FEV1 (% predicted)	78.0 ± 21.64	78.0 ± 20.81	77.0 ± 22.26	0.084
FEV1/FVC ratio (%)	84.20 ± 9.04	85.15 ± 8.57	83.41 ± 9.40	0.30
TLC (% predicted)	78.50 ± 17.07	83.0 ± 15.48	71.0 ± 17.00	0.200
Muscle variables				
Hand grip strength (kg), mean ± SD	26.49 ± 9.57	20.73 ± 5.90	30.86 ± 9.52	0.016
ASMMI (kg/m^2^), mean ± SD	6.51 ± 1.22	5.71 ± 0.97	7.12 ± 1.02	< 0.001
Low muscle strength, *n* (%)	44 (28.8%)	13 (8.4%)	31 (20.1)	0.03
Low muscle mass, *n* (%)	68 (44.4%)	25 (16.2%)	43 (27.9%)	0.16
Sarcopenia prevalence, *n* (%)	29 (19.1%)	10 (15.2%)	19 (22.1%)	0.06

Corrected Table 1:

**Table:** 

Variable	Total (*n* = 155)	Women (*n* = 67)	Men (*n* = 88)	*p*
Age (years), mean ± SD	48.7 ± 13.3	44.6 ± 14.3	51.9 ± 11.5	< 0.001
BMI (kg/m^2^), mean ± SD	22.9 ± 4.03	21.36 ± 3.46	24.2 ± 3.99	< 0.001
ADL, mean ± SD	5.84 ± 0.62	5.88 ± 0.48	5.81 ± 0.71	0.46
Primary condition, *n* (%)				0.01
Cystic fibrosis	46 (30.1%)	28 (42.4%)	18 (20.7%)	
Restrictive lung diseases	34 (22.2%)	16 (24.2%)	18 (20.7%)	
Obstructive lung diseases	24 (15.7%)	5 (7.6%)	19 (21.8%)	
Miscellaneous	41 (26.8%)	13 (19.7%)	28 (32.2%)	
Vascular diseases	8 (5.2%)	4 (4.6%)	4 (6.1%)	
Time since transplant (months), mean ± SD	40.01 ± 80.71	40.94 ± 52.76	39.31 ± 97.05	0.890
Total medications taken, mean ± SD	15.05 ± 3.86	14.87 ± 3.88	15.18 ± 3.87	0.614
Duration of corticosteroid therapy (months), mean ± SD	30.19 ± 47.98	38.51 ± 61.60	23.84 ± 33.12	0.061
CIRS‐CI, mean ± SD	4.05 ± 1.67	4.00 ± 1.52	4.08 ± 1.79	0.770
Pulmonary volumes, mean ± SD				
VC (% predicted)	78.5 ± 20.27	82.0 ± 20.61	73.0 ± 19.49	0.094
FVC (% predicted)	75.94 ± 20.52	78.40 ± 20.13	73.97 ± 20.74	0.013
FEV1 (% predicted)	78.0 ± 21.64	78.0 ± 20.81	77.0 ± 22.26	0.084
FEV1/FVC ratio (%)	84.20 ± 9.04	85.15 ± 8.57	83.41 ± 9.40	0.30
TLC (% predicted)	78.50 ± 17.07	83.0 ± 15.48	71.0 ± 17.00	0.200
Muscle variables				
Hand grip strength (kg), mean ± SD	26.49 ± 9.57	20.73 ± 5.90	30.86 ± 9.52	0.016
ASMMI (kg/m^2^), mean ± SD	6.51 ± 1.22	5.71 ± 0.97	7.12 ± 1.02	< 0.001
Low muscle strength, *n* (%)	44 (28.8%)	13 (8.4%)	31 (20.1)	0.03
Low muscle mass, *n* (%)	68 (44.4%)	25 (16.2%)	43 (27.9%)	0.16
Sarcopenia prevalence, *n* (%)	29 (19.1%)	10 (15.2%)	19 (22.1%)	0.06

In Table 2, the femoral neck BMD value for the total sample was also incorrect: 0.92 ± 0.49 should read 0.66 ± 0.15.

Incorrect Table 2:

**Table:** 

Variable	Total (*n* = 155)	Women (*n* = 67)	Men (*n* = 88)	*p*
Pretransplant evaluation, *n* (%)				
Vitamin D/calcium supplementation	102 (62.5%)	48 (72.7%)	54 (61.4%)	0.09
Antiresorptive therapy	29 (18.7%)	9 (13.4%)	20 (22.7%)	0.10
Anabolic therapy	2 (1.3%)	0	2 (2.3%)	0.45
Osteoporosis diagnosis	34 (21.9%)	13 (19.4%)	21 (23.9%)	0.01
Posttransplant evaluation				
Densitometric values, mean ± SD				
*T*‐score lumbar	−1.71 ± 1.41	−1.73 ± 1.36	−1.69 ± 1.46	0.86
*T*‐score femur neck	−1.92 ± 1.09	−2.22 ± 0.94	−1.70 ± 1.14	< 0.001
T‐score total hip	−1.53 ± 1.01	−1.91 ± 0.94	−1.25 ± 0.98	< 0.001
BMD lumbar	0.92 ± 0.49	0.94 ± 0.73	0.90 ± 0.16	0.58
BMD femur neck	0.92 ± 0.49	0.62 ± 0.13	0.69 ± 0.15	< 0.001
BMD total hip	0.77 ± 0.18	0.71 ± 0.14	0.83 ± 0.17	< 0.001
Diagnosis, *n* (%)				0.10
Osteopenia	29 (18.7%)	9 (13.4%)	20 (22.7%)	
Osteoporosis	82 (52.9%)	25 (37.3%)	57 (64.7%)	
At least one vertebral fracture, *n* (%)	52 (33.5%)	14 (20.9%)	38 (43.2%)	0.005
Anti‐resorptive therapy, *n* (%)	109 (70.3%)	41 (61.2%)	68 (77.3%)	0.003
Anabolic therapy, *n* (%)	11 (7.1%)	2 (3.0%)	9 (10.2%)	0.003

Corrected Table 2:

**Table:** 

Variable	Total (*n* = 155)	Women (*n* = 67)	Men (*n* = 88)	*p*
Pretransplant evaluation, *n* (%)				
Vitamin D/calcium supplementation	102 (62.5%)	48 (72.7%)	54 (61.4%)	0.09
Anti‐resorptive therapy	29 (18.7%)	9 (13.4%)	20 (22.7%)	0.10
Anabolic therapy	2 (1.3%)	0	2 (2.3%)	0.45
Osteoporosis diagnosis	34 (21.9%)	13 (19.4%)	21 (23.9%)	0.01
Posttransplant evaluation				
Densitometric values, mean ± SD				
*T*‐score lumbar	−1.71 ± 1.41	−1.73 ± 1.36	−1.69 ± 1.46	0.86
*T*‐score femur neck	−1.92 ± 1.09	−2.22 ± 0.94	−1.70 ± 1.14	< 0.001
T‐score total hip	−1.53 ± 1.01	−1.91 ± 0.94	−1.25 ± 0.98	< 0.001
BMD lumbar	0.92 ± 0.49	0.94 ± 0.73	0.90 ± 0.16	0.58
BMD femur neck	0.66 ± 0.15	0.62 ± 0.13	0.69 ± 0.15	< 0.001
BMD total hip	0.77 ± 0.18	0.71 ± 0.14	0.83 ± 0.17	< 0.001
Diagnosis, *n* (%)				0.10
Osteopenia	29 (18.7%)	9 (13.4%)	20 (22.7%)	
Osteoporosis	82 (52.9%)	25 (37.3%)	57 (64.7%)	
At least one vertebral fracture, *n* (%)	52 (33.5%)	14 (20.9%)	38 (43.2%)	0.005
Anti‐resorptive therapy, *n* (%)	109 (70.3%)	41 (61.2%)	68 (77.3%)	0.003
Anabolic therapy, *n* (%)	11 (7.1%)	2 (3.0%)	9 (10.2%)	0.003

These corrections do not affect the results or the overall conclusions of the study.

We apologize for this error.

