# Supplementary material for: Sex Differences in Muscle–Respiratory Function Relationship in Lung Transplant Patients: A Longitudinal Study
Source: J Cachexia Sarcopenia Muscle. 2026 Mar 5;17(2):e70244. doi: 10.1002/jcsm.70244 (PMC12961349; doi:10.1002/jcsm.70244)
Supplement: Supplementary file 1 — Figure S1: Violin plots showing the distribution of pulmonary function parameters over time. Table S1: Linear mixed models for changes in lung volumes over time in patients with cystic fibrosis, stratified by gender. [file JCSM-17-e70244-s001.docx]

**Supplementary references:**

[S1] C. Caffarelli *et al.*, “Bone fragility and sarcoidosis: An underestimated relationship.,” *Front. Med.*, vol. 9, p. 1026028, 2022, doi: 10.3389/fmed.2022.1026028.

[S2] A. Anabtawi, T. Le, M. Putman, V. Tangpricha, and M. L. Bianchi, “Cystic fibrosis bone disease: Pathophysiology, assessment and prognostic implications.,” *J. Cyst. Fibros. Off. J. Eur. Cyst. Fibros. Soc.*, vol. 18 Suppl 2, pp. S48–S55, Oct. 2019, doi: 10.1016/j.jcf.2019.08.018.

[S3] B. S. LINN, M. W. LINN, and L. GUREL, “CUMULATIVE ILLNESS RATING SCALE,” *J. Am. Geriatr. Soc.*, 1968, doi: 10.1111/j.1532-5415.1968.tb02103.x.

[S4]S. Katz, “Assessing self-maintenance: Activities of daily living, mobility, and instrumental activities of daily living,” *J. Am. Geriatr. Soc.*, vol. 31, no. 12, pp. 721–727, 1983, doi: 10.1111/j.1532-5415.1983.tb03391.x.

[S5]J. A. Kanis, L. J. 3rd Melton, C. Christiansen, C. C. Johnston, and N. Khaltaev, “The diagnosis of osteoporosis.,” *J. bone Miner. Res. Off. J. Am. Soc. Bone Miner. Res.*, vol. 9, no. 8, pp. 1137–1141, Aug. 1994, doi: 10.1002/jbmr.5650090802.

[S6]R. H. J. A. Slart *et al.*, “Updated practice guideline for dual-energy X-ray absorptiometry (DXA),” *Eur. J. Nucl. Med. Mol. Imaging*, vol. 52, no. 2, pp. 539–563, 2025, doi: 10.1007/s00259-024-06912-6.

[S7]S. Baim *et al.*, “Official Positions of the International Society for Clinical Densitometry and executive summary of the 2007 ISCD Position Development Conference.,” *J. Clin. Densitom. Off. J. Int. Soc. Clin. Densitom.*, vol. 11, no. 1, pp. 75–91, 2008, doi: 10.1016/j.jocd.2007.12.007.

[S8]H. K. Genant, C. Y. Wu, C. van Kuijk, and M. C. Nevitt, “Vertebral fracture assessment using a semiquantitative technique,” *J. Bone Miner. Res.*, 1993, doi: 10.1002/jbmr.5650080915.

[S9]A. R. Sousa-Santos and T. F. Amaral, “Differences in handgrip strength protocols to identify sarcopenia and frailty - a systematic review.,” *BMC Geriatr.*, vol. 17, no. 1, p. 238, Oct. 2017, doi: 10.1186/s12877-017-0625-y.

[S10]J. A. Kanis, “Diagnosis of osteoporosis.,” *Osteoporos. Int. a J. Establ. as result Coop. between Eur. Found. Osteoporos. Natl. Osteoporos. Found. USA*, vol. 7 Suppl 3, pp. S108-16, 1997, doi: 10.1007/BF03194355.

[S11]S. Ferrari *et al.*, “Osteoporosis in young adults: Pathophysiology, diagnosis, and management,” *Osteoporosis International*. 2012, doi: 10.1007/s00198-012-2030-x.

[S12] H.-J. Kim *et al.*, “Glucocorticoids suppress bone formation via the osteoclast.,” *J. Clin. Invest.*, vol. 116, no. 8, pp. 2152–2160, Aug. 2006, doi: 10.1172/JCI28084

[S13] M. R. Rubin and J. P. Bilezikian, “Clinical Review 151: The role of parathyroid hormone in the pathogenesis of glucocorticoid-induced osteoporosis: A re-examination of the evidence,” *Journal of Clinical Endocrinology and Metabolism*. 2002, doi: 10.1210/jc.2002-012101.

[S14]M. K. Balci *et al.*, “Osteoporosis in Lung Transplantation Candidates: Association With 6-minute Walking Test and Body Mass Index,” *Transplant. Proc.*, vol. 48, no. 6, pp. 2147–2151, 2016, doi: 10.1016/j.transproceed.2016.02.074.

[S15]W. C. Lakey, S. Spratt, E. N. Vinson, D. Gesty-Palmer, T. Weber, and S. Palmer, “Osteoporosis in lung transplant candidates compared to matched healthy controls.,” *Clin. Transplant.*, vol. 25, no. 3, pp. 426–435, 2011, doi: 10.1111/j.1399-0012.2010.01263.x.

[S16]M. S. Arenas-de Larriva, J. M. Vaquero-Barrios, J. Redel-Montero, and F. Santos-Luna, “Bone mineral density in lung transplant candidates.,” *Transplant. Proc.*, vol. 42, no. 8, pp. 3208–3210, Oct. 2010, doi: 10.1016/j.transproceed.2010.05.058.

[S17]C. Caffarelli *et al.*, “Timing of Osteoporotic Vertebral Fractures in Lung and Heart Transplantation: A Longitudinal Study.,” *J. Clin. Med.*, vol. 9, no. 9, Sep. 2020, doi: 10.3390/jcm9092941.

[S18]T. Munhoz da Rocha Lemos Costa *et al.*, “Bone mineral density and vertebral fractures and their relationship with pulmonary dysfunction in patients with chronic obstructive pulmonary disease,” *Osteoporos. Int. 2018 2911*, vol. 29, no. 11, pp. 2537–2543, Jul. 2018, doi: 10.1007/S00198-018-4643-1.

[S19]E. G. Culham, H. A. I. Jimenez, and C. E. King, “Thoracic kyphosis, rib mobility, and lung volumes in normal women and women with osteoporosis,” *Spine (Phila. Pa. 1976).*, 1994, doi: 10.1097/00007632-199405310-00010.

[S20]V. Hoang, G. W. Li, C. C. Kao, G. Dronavalli, and A. D. Parulekar, “Determinants of pre-transplantation pectoralis muscle area (PMA) and post-transplantation change in PMA in lung transplant recipients.,” *Clin. Transplant.*, vol. 31, no. 3, Mar. 2017, doi: 10.1111/ctr.12897.

[S21]P. Calella, G. Valerio, M. Brodlie, L. M. Donini, and M. Siervo, “Cystic fibrosis, body composition, and health outcomes: a systematic review.,” *Nutrition*, vol. 55–56, pp. 131–139, Nov. 2018, doi: 10.1016/j.nut.2018.03.052.

**Supplementary Figure 1. Violin plots showing the distribution of pulmonary function parameters over time.**


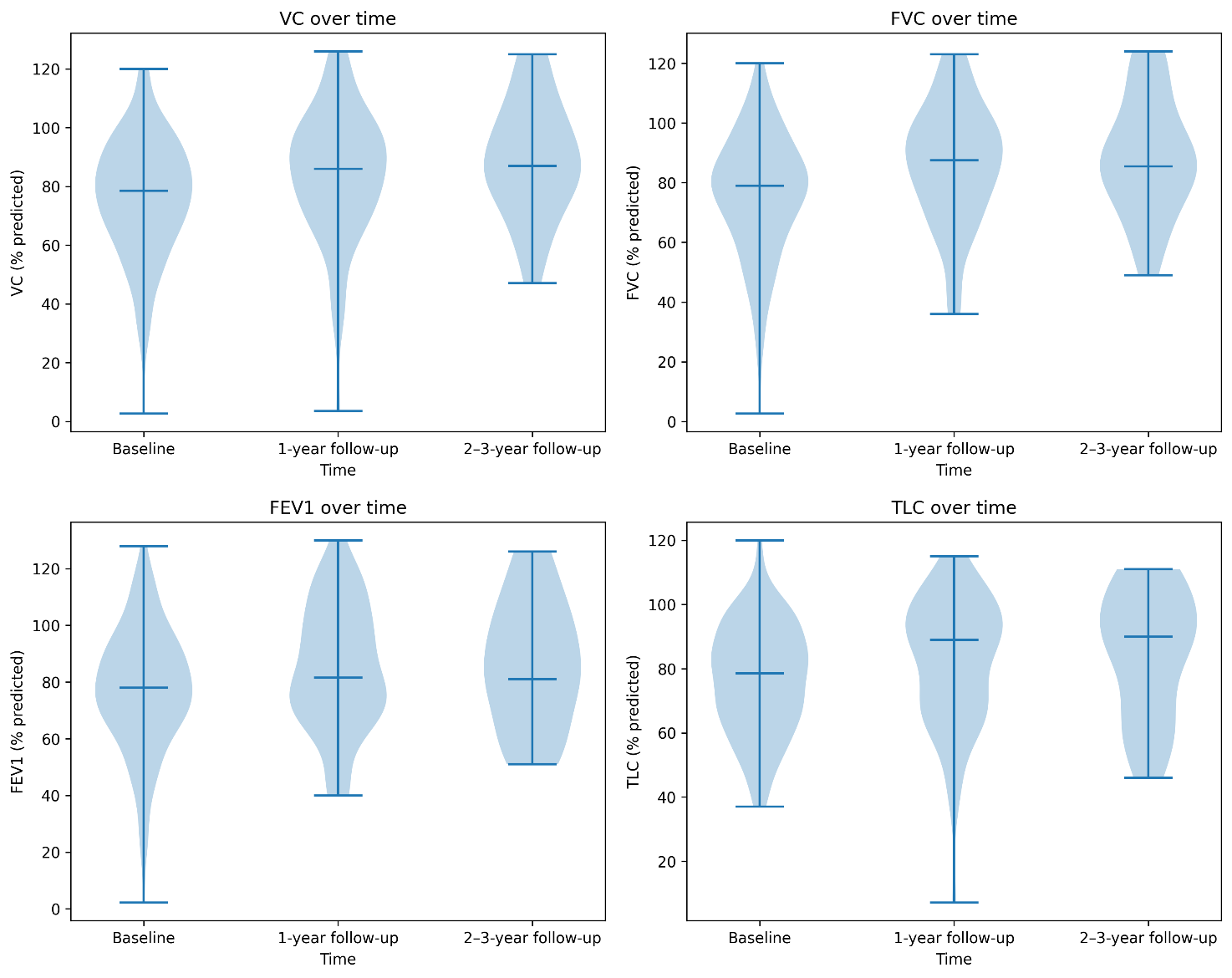


Panels show (A) VC, (B) FVC, (C) FEV1, and (D) TLC expressed as percentage of predicted values at baseline, 1-, and 2-3-year follow-up. The width of each violin represents data density, and the horizontal line indicates the median. Abbreviations: VC, vital capacity; FVC, forced vital capacity; FEV1, forced expiratory volume in 1 second; TLC, total lung capacity.

**Supplementary Table 1. Linear Mixed Models for Changes in Lung Volumes Over Time in Patients with Cystic Fibrosis, Stratified by Gender.**

| **Variable** | **Men** | | | **Women** | | |
| --- | --- | --- | --- | --- | --- | --- |
|  | **Model 1** | **Model 2** | **Model 3** | **Model 1** | **Model 2** | **Model 3** |
|  | **β coefficient (95% CI), p-value** | **β coefficient (95% CI), p-value** | **β coefficient (95% CI), p-value** | **β coefficient (95% CI), p-value** | **β coefficient (95% CI), p-value** | **β coefficient (95% CI), p-value** |
| **VC** |  |  |  |  |  |  |
| HG*time | 0.004 (-0.14; 0.15),  p = 0.94 | 0.01 (-0.15; 0.17),  p = 0.91 | -0.05 (-0.22; 0.10),  p = 0.41 | 0.22 (-0.29; 0.75),  p = 0.39 | 0.15 (-0.37; 0.68),  p = 0.56 | 0.05 (-0.48; 0.59),  p = 0.84 |
| ASMMI*time | 1.00 (-19.63; 6.46),  p = 0.23 | 0.96 (-0.93; 2.86),  p = 0.28 | 1.00 (-0.94; 2.94),  p = 0.25 | 1.18 (-0.43; 2.78),  p = 0.14 | 0.89 (-0.77; 2.55),  p = 0.28 | 0.73 (-1.02; 2.48),  p = 0.40 |
| **FVC** |  |  |  |  |  |  |
| HG*time | -0.03 (-0.17; 0.11),  p = 0.64 | -0.03 (-0.17; 0.12),  p = 0.70 | -0.12 (-0.27; 0.04),  p = 0.11 | -0.06 (-0.51; 0.39),  p = 0.80 | -0.01 (-0.51; 0.48),  p = 0.95 | -0.04 (-0.56; 0.48),  p = 0.88 |
| ASMMI*time | 0.68 (-1.05; 2.41),  p = 0.15 | 0.55 (-1.34; 2.43),  p = 0.52 | 0.83 (-1.22; 2.88),  p = 0.37 | 0.82 (-0.53; 2.17),  p = 0.22 | 0.87 (-0.64; 2.37),  p = 0.24 | 1.04 (-0.57; 2.64),  p = 0.19 |
| **FEV1** |  |  |  |  |  |  |
| HG*time | 0.02 (-0.18; 0.22),  p = 0.82 | 0.03 (-0.18; 0.25),  p = 0.75 | -0.06 (-0.27; 0.16),  p = 0.56 | 0.35 (-0.28; 0.98),  p = 0.27 | 0.28 (-0.36; 0.93),  p = 0.37 | 0.20 (-0.47; 0.87),  p = 0.54 |
| ASMMI*time | 1.60 (-0.62; 3.82),  p = 0.14 | 1.51 (-0.93; 3.95),  p = 0.19 | 1.75 (-0.49; 4.00),  p = 0.10 | 0.98 (-1.02; 2.98),  p = 0.32 | 0.71 (-1.37; 2.79),  p = 0.49 | 0.63 (-1.58; 2.84),  p = 0.56 |
| **TLC** |  |  |  |  |  |  |
| HG*time | 0.20 (-0.15; 0.56),  p = 0.23 | 0.22 (-0.15; 0.59),  p = 0.21 | 0.38 (-1.65; 1.65),  p = 0.81 | **0.45 (0.02; 0.89),  p = 0.04** | **0.41 (0.02; 0.84),  p = 0.04** | **0.38 (0.07; 0.84),  p = 0.04** |
| ASMMI*time | -1.42 (-6.04; 3.21),  p = 0.51 | -2.00 (-6.71; 2.72),  p = 0.37 | -1.97 (-7.73; 3.78),  p = 0.48 | 0.70 (-0.73; 2.14),  p = 0.32 | 0.58 (-0.86; 2.03),  p = 0.42 | 0.68 (-0.90; 2.25),  p = 0.39 |

**Abbreviations**: VC, Tidal Volume; FVC, Forced Vital Capacity; FEV1, Forced Expiratory Volume in 1 Second; TLC, Total Lung Capacity; ASMMI, Appendicular Skeletal Muscle Mass Index; HGS, Hand Grip Strength Test.

*Model 1* includes age. *Model 2* also includes functional capacities and steroid dosage. *Model 3* includes the presence of vertebral fractures, CIRS-CI, and time since transplant. Significant results (p < 0.05) are shown in bold.
